# Temporal Characterization of Homology-Independent Centromere Coupling in Meiotic Prophase

**DOI:** 10.1371/journal.pone.0010336

**Published:** 2010-04-23

**Authors:** David Obeso, Dean S. Dawson

**Affiliations:** 1 Cell Cycle and Cancer Biology, Oklahoma Medical Research Foundation, Oklahoma City, Oklahoma, United States of America; 2 Department of Cell Biology, University of Oklahoma Health Sciences Center, Oklahoma City, Oklahoma, United States of America; National Cancer Institute, United States of America

## Abstract

**Background:**

Over the past thirty years several reports of the pairing or association of non-homologous centromeres during meiotic prophase have appeared in the literature. Recently, the homology-independent pairwise association of centromeres, termed centromere coupling, was also reported in budding yeast. It seems paradoxical that centromeres would pair with non-homologous partners during a process intended to align homologous chromosomes, yet the conservation of this phenomenon across a wide range of species suggests it may play an important role in meiosis.

**Principal Findings:**

To better define the role of this phenomenon in budding yeast, experiments were preformed to place centromere coupling within the context of landmark meiotic events. Soon after the initiation of the meiotic program, centromeres were found to re-organize from a single cluster into non-homologous couples. Centromere coupling is detected as soon as chromosome replication is finished and persists while the recombination protein Dmc1 is loaded onto the chromosomes, suggesting that centromere coupling persists through the time of double strand break formation. In the absence of the synaptonemal complex component, Zip1, centromere coupling was undetectable, at all times examined, confirming the essential role of this protein on this process. Finally, the timely release of centromere coupling depends on the recombination-initiating enzyme, Spo11, suggesting a connection between events in homologous pairing/recombination and the regulation of centromere coupling.

**Conclusions:**

Based on our results we propose a role for centromere coupling in blocking interactions between homologous centromeres as recombination initiation is taking place.

## Introduction

In order to correctly segregate from one another at meiosis I, homologous chromosomes need first to become associated. In most organisms the selection of the segregation partners is based on sequence homology and recombination along the chromosome arms, and not the centromeric regions, as manipulation of homologous chromosomes so that they have non-homologous centromeres has little deleterious effect on their segregation behavior [1], [2].

In many organisms, including budding yeast, crossing-over is repressed near the centromeres [3]. This crossover repression is beneficial, as crossovers in centromere-proximal regions of budding yeast, *Drosophila* and humans are associated with elevated levels of chromosome segregation errors in meiosis [4], [5].

Over thirty years ago experiments in onion meiocytes revealed that the centromeres became organized in non-homologous pairs or small groups, prior to the period of homologous chromosome synapsis [6]. Subsequent similar observations in other species showed that the association of non-homologous centromeres during meiotic prophase is a widespread phenomenon (reviewed in [7]). Recently the phenomenon was reported in budding yeast where it was demonstrated that the centromeres of the thirty-two chromosomes form sixteen pairwise associations, usually between non-homologous partners, at a period prior to the alignment of homologous chromosomes [8]. To differentiate this homology-independent centromeric association from the regular pairing between homologous chromosomes this phenomenon has been referred to as centromere coupling [8]. In budding yeast, centromere coupling has been shown to require the protein Zip1 [8]. Zip1 constitutes the central element of the synaptonemal complex (SC), and is necessary to bridge the lateral elements that assemble along the cores of the chromosomes, thus zippering the homologous partners together [9], [10]. The exact role of Zip1 in centromere coupling is not known but it is tempting to think that it may perform a similar role as it plays for SC formation, directly bringing non-homologous centromeres together by its ability to self-associate.

Why, in many organisms, do centromeres become coupled with non-homologous partners when meiosis has evolved a series of elaborate mechanisms to ensure the pairing of homologous chromosomes? To help address this question we performed time course experiments to place the period of centromere coupling within the context of other meiotic landmark events, an approach that has not been taken by previous studies. The results favor a new model for the function of centromere coupling.

## Results

### Centromere coupling dynamics in WT cells

**Figure 1:**
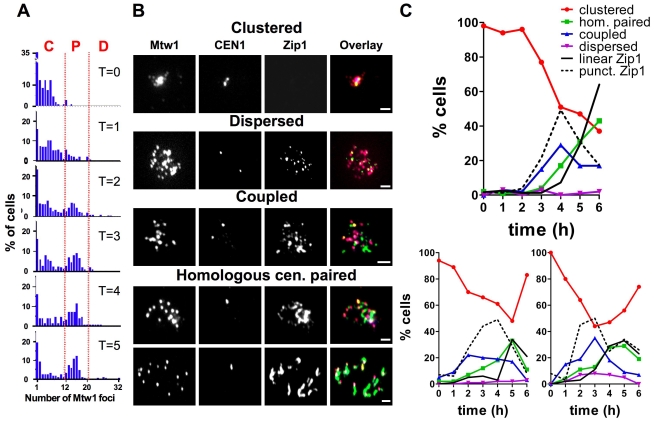
Dynamics of centromere coupling. Meiotic cells (DDO45 and DDO46) were evaluated for the behavior of centromeres by indirect immunofluorescence observation of kinetochores (Mtw1-13XMYC), a pair of homologous centromeres (GFP-tagged *CEN1*) and Zip1, in chromosome spreads. (A) The number of Mtw1-13XMYC foci was determined at each time point (n>100 for each time point). C: clustered (<12 Mtw1 foci); P: paired (either between homologs or non homologs: 12–20 Mtw1 foci); D: dispersed (>20 Mtw1 foci) (B) Examples of categories of centromere organization: clustered, dispersed, non-homologous coupled (12–20 Mtw1 foci, separate *CEN1*-GFP foci), and homologous paired (12–20 Mtw1 foci, one CEN1-GFP focus). Mtw1: red; *CEN1*: yellow; Zip1: green. Scale bar: 2µm (C) The proportion of chromosome spreads in each category (DDO45 and DDO46; n>50 for each time point). Three iterations of this experiment can be seen. The percentages of cells with punctate and linear Zip1 staining are shown as a reference of meiotic progression in each individual experiment.

To characterize centromere coupling we compared the kinetics of the formation of non-homologous and homologous centromere pairs in budding yeast cells harvested at intervals from cultures induced to enter meiosis. The kinetochore protein Mtw1 was epitope-tagged, allowing us to score the number of kinetochore foci in spread nuclei. Diploid yeast cells have sixteen homologous chromosome pairs. If the centromeres of the chromosomes are arranged in pairs, each nucleus will exhibit sixteen Mtw1 foci. As described previously [11], [12] we observed that the centromeres are tightly clustered early in meiosis. Chromosome spreads typically exhibited fewer than ten Mtw1 foci at the time of meiotic induction (Fig. 1A, T = 0), about two hours later a population appeared that exhibited about sixteen foci (Fig. 1A, T = 2–T = 5), consistent with pairing of the centromeres. In order to quantify the emergence and disappearance of different types of centromere organization we categorized nuclei with fewer than twelve foci as “clustered”, nuclei with twelve to twenty foci as “paired” and nuclei with more than twenty as “dispersed” (Fig. 1A).

Cells with about sixteen Mtw1 foci could be engaged a period of centromere coupling or homologous alignment of the chromosomes (or a transition between the two). To distinguish between these possibilities both copies of chromosome *I* were GFP-tagged at their centromeres (*CEN1*) [13]. When homologous chromosomes become synapsed, the GFP-tags of the two *CEN1's* will be juxtaposed; yielding one single or two closely paired fluorescent dots that co-localize with a single Mtw1 focus. Conversely, centromere coupling has been shown to occur largely between non-homologous partners [8] so during a period of centromere coupling, the two *CEN1* GFP-tags will usually be associated with different Mtw1 foci. In early time points, in chromosome spreads with 12–20 Mtw1 foci, the two copies of *CEN1* (GFP) were nearly always associated with different Mtw1 foci (Figs. 1B and 1C). Homologous centromere pairing emerged about an hour after coupling is first detected (Figs. 1B and 1C). In samples harvested from later time points the proportion of cells with homologous centromere pairing increased as the proportion of cells with centromere coupling decreased (Figs. 1B and 1C). This experiment shows that centromeres go through transitions; from clustered, to non homologous coupling, to homologous alignment as cells proceed through meiotic prophase, a progression similar to that described for centromeres in wheat (reviewed in [7]). Throughout the time course, spreads with more than twenty Mtw1 foci (dispersed) were rare (Figs. 1B and 1C). In our experiments the exact timing of meiosis varied somewhat between isogenic strains and also in independent repetitions using same strain (Fig 1C). Thus, we measured SC formation/disassembly during the multiple time courses (Fig 1C, linear Zip1). Despite small variations from one repetition to the next, the relative order of centromere coupling with respect to pairing and the SC formation is always identical (Fig 1C). In some time courses the proportion of clustered cells increased at late time points (Fig. 1C, red lines). This occurred in samples that entered meiosis more quickly after the switch to meiosis inducing medium (Fig 1C). The increase in clustered cells, is probably attributable in part to the recently described re-clustering of the centromeres at the side-by-side spindle pole bodies right after pachytene [14], and also to the fact that once cells became bi-nucleate in these experiments, they were excluded from the analysis. One population of mononucleate cells are those cells that do not progress through the meiotic program and never form asci (typically about 20% of the cells with the strains used here). These cells remain as mononucleate cells with clustered centromeres and no Zip1 staining, and represent an ever-growing proportion of the mononucleate cell population as cells that have entered the meiotic program become multinucleate.

### Centromere coupling and the appearance of Zip1

**Figure 2:**
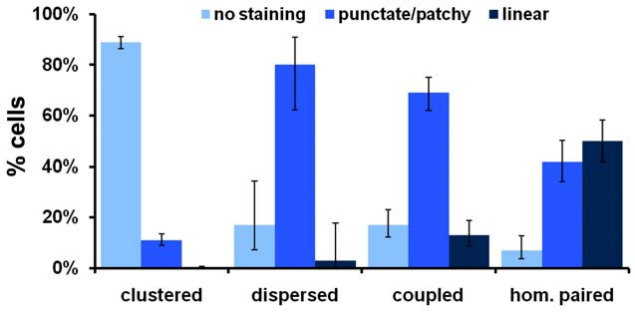
Zip1 staining pattern on cells classified by their centromere behavior. Cells from the time courses shown in Figure 1 were classified according to their pattern of Zip1 staining. Zip1 categories: absent (as in the clustered example, Fig 1 B), punctate/patchy (an example of punctate staining in the Dispersed example, Fig. 1 B, examples of patchy staining are the Coupled and top row of Homologous examples in Fig. 1 B) or linear (an example is the bottom row of Homologous in Fig. 1 B). Clustered (n = 726), dispersed (n = 30), non-homologous paired (n = 186) and homologous paired (n = 139). Error bars: 95% confidence intervals.

Centromere coupling has been shown to require the SC protein Zip1 [8]. We therefore compared the timing of centromere coupling with the timing of Zip1 loading onto the chromosomes. 90% (n = 726) of chromosome spreads with clustered centromeres showed no Zip1 staining (Fig. 1B and Fig. 2). The detection of centromere coupling and localization of Zip1 to the chromosomes appeared simultaneously (Fig 1C, compare “coupled” and “punct. Zip1”). About 75% of cells undergoing centromere coupling exhibited numerous Zip1 punctate foci (Fig. 1B and 2). The initial study of centromere coupling reported that in chromosome spreads exhibiting centromere coupling, Zip1 foci and kinetochore pairs occur in approximately equal numbers (about sixteen) and exhibit a high level of co-localization [8]. In contrast, in spreads that exhibited punctate Zip1 staining and were undergoing centromere coupling, we observed considerable variation in the total number of Zip1 foci (average 22.4 +/− S.D. 7.2, n = 20 spreads). This result is consistent with previous studies of Zip1 association with chromosomes in early meiosis [15], [16]. Some of the Zip1 foci we detect co-localize with kinetochores (Mtw1-13XMYC) but most do not (29.0% co-localization of Zip1 foci to Mtw1 foci, +/− S.D. 12.0, n = 20 spreads) (Fig. 1 B), a result similar to that previously observed by Bardhan et al [17]. The punctate Zip1 signals detected at centromeres in these experiments were not of uniform intensity suggesting that different amounts of Zip1 are localized to different centromeres. The modest Zip1/Mtw1 co-localization reported here does not necessarily mean that Zip1 is not located at all centromere couples and could be explained if small numbers of Zip1 molecules (not detectable by our staining) are sufficient for centromere coupling. The result also shows that Zip1 localizes to other chromosomal loci besides centromeres in early meiosis.

Finally, about 10% of the cells that were engaged in centromere coupling (*CEN1*'s associated with separate Mtw1 foci) exhibited linear SC (Fig. 2) demonstrating that, in some cells, considerable synapsis occurs before the homologous *CEN1*'s become aligned - consistent with the model that the alignment of chromosome arms generally precedes the alignment of homologous centromeres. As expected, almost all cells with paired homologous *CEN1*'s showed positive Zip1 staining: 50% linear staining, typical of synapsed chromosomes [9], and 42% punctate or patchy staining, corresponding to SC assembly and SC disassembly stages.

### Centromere coupling dynamics in *spo11* and *zip1* mutants

**Figure 3:**
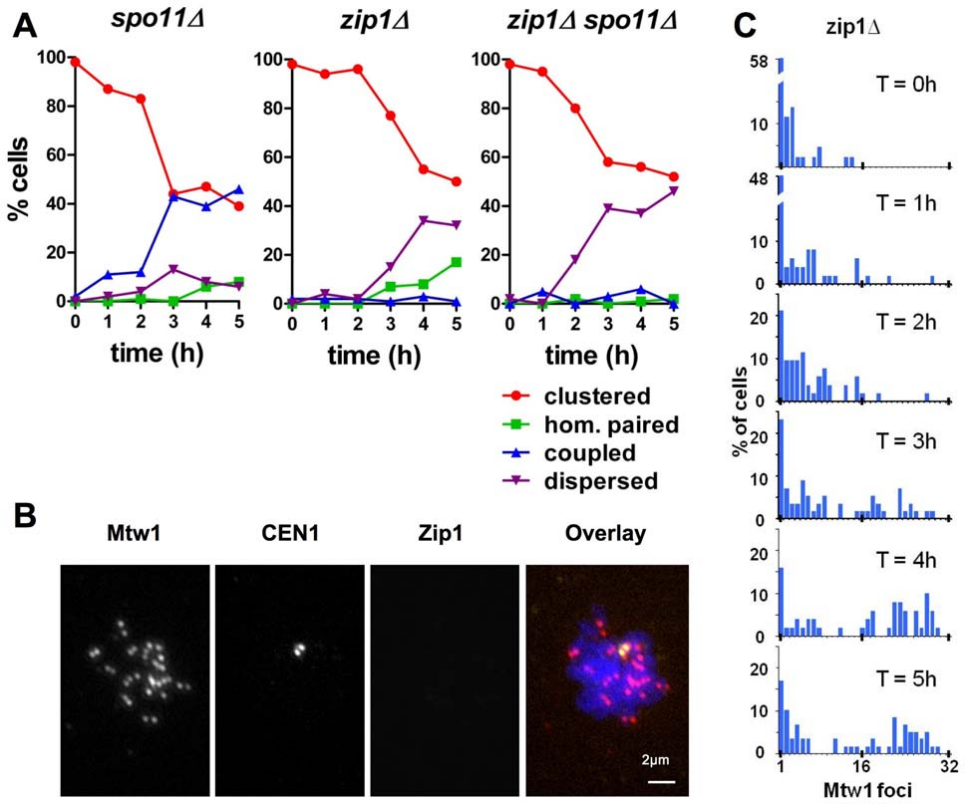
Dynamics of centromere coupling in *spo11* and *zip1* deletion mutants. Meiotic time course experiments were performed as described in Figure 1, to evaluate centomere coupling in *spo11* and *zip1* deletion mutants. (A) *spo11Δ* (DDO60), *zip1Δ* (DDO55) and *zip1Δ spo11Δ* (DDO56) strains were evaluated for their patterns of centromere organization (n>50 per time point). (B) A representative *zip1*Δ cell (from T = 5 hours) with dispersed centromeres. Scale bar: 2µm. (C) The number of Mtw1 foci in a *zip1Δ* deletion strain (DDO55) was evaluated by indirect immune-fluorescence (n>45 for each time point).

Centromere coupling was originally noted in *spo11Δ* mutants [8]. The ease of obtaining chromosome spreads in the time point used in those experiments suggests that cells might persist in the centromere coupling stage in *spo11Δ* mutants. To test this, we evaluated early centromere behavior in *spo11Δ* mutants, which exhibit severe defects in the pairing and synapsis of homologous chromosome arms [18], [19]. Whereas in the wild-type strain, the frequency of cells exhibiting centromere coupling begins to diminish by three/four hours in meiosis (Fig. 1 C), in *spo11Δ* mutants, the proportion of cells with non-homologous coupled centromeres continues to rise and persists at all times evaluated (Fig. 3A, *spo11*Δ). Thus, the timely release from centromere coupling depends on the presence of Spo11p.

Previous work (single time point experiments) revealed that *zip1* mutant cells in late meiotic prophase do not exhibit centromere coupling, suggesting that Zip1 is necessary either for the initiation of centromere coupling or its persistence [8]. To distinguish between these possibilities we evaluated centromere coupling in cells harvested from multiple time points following meiotic induction (Fig. 3A, *zip1*Δ). A requirement for Zip1 in initiation of coupling would predict that coupling would not be observed at any time point, whereas a role in maintenance could result in the observation of centromere coupling at early time points that does not persist until late meiotic prophase. We did not detect centromere coupling in *zip1Δ* cells at any time point evaluated. Instead, concomitant with the exit from centromere clustering we observed the appearance of chromosome spreads with dispersed centromeres (more than 20 Mtw1 foci) (Fig. 3A, *zip1*Δ and 3C). Some of the dispersed spreads we observe in *zip1Δ* mutants reflect a role for Zip1 in pairing the centromeres of homologous chromosomes in late prophase [14], [19], [20], [21]. In the absence of *ZIP1*, homologs become aligned, but because Zip1 is necessary for tight pairing of homologous centromeres in late meiotic prophase, their centromeres are often slightly separated and thus visualized as side-by-side Mtw1 foci [14], [20], [21]. Because side-by-side foci are scored as two separate foci in our assay, this yields higher counts of Mtw1 foci. Consistent with this, in the *zip1Δ* strain the “dispersed” spreads from the four and five hour time points often feature side-by-side *CEN1* GFP dots associated with separate but adjacent Mtw1 foci (37% at T = 4 hr, 67% at T = 5 hr) (Fig. 3B). The model that coupling requires Zip1 also predicts that in a *spo11Δ zip1Δ* double mutant, cells exhibiting dispersed, individual, centromeres will accumulate, since the loss of Zip1 should eliminate the centromere coupling and loss of Spo11 should eliminate homologous alignment. This was in fact observed (Fig. 3A, *spo11Δ zip1Δ*); *spo11Δ zip1Δ* double mutants transition from the clustered stage to a dispersed organization of centromeres.

### Centromere coupling is co-incident with early events in homologous recombination

Determining the placement of centromere coupling among the landmark events of early meiosis might provide clues to the function of non-homologous centromere associations. Thus we compared the timing of centromere coupling to pre-meiotic DNA replication and the early stages of homologous meiotic recombination.

**Figure 4:**
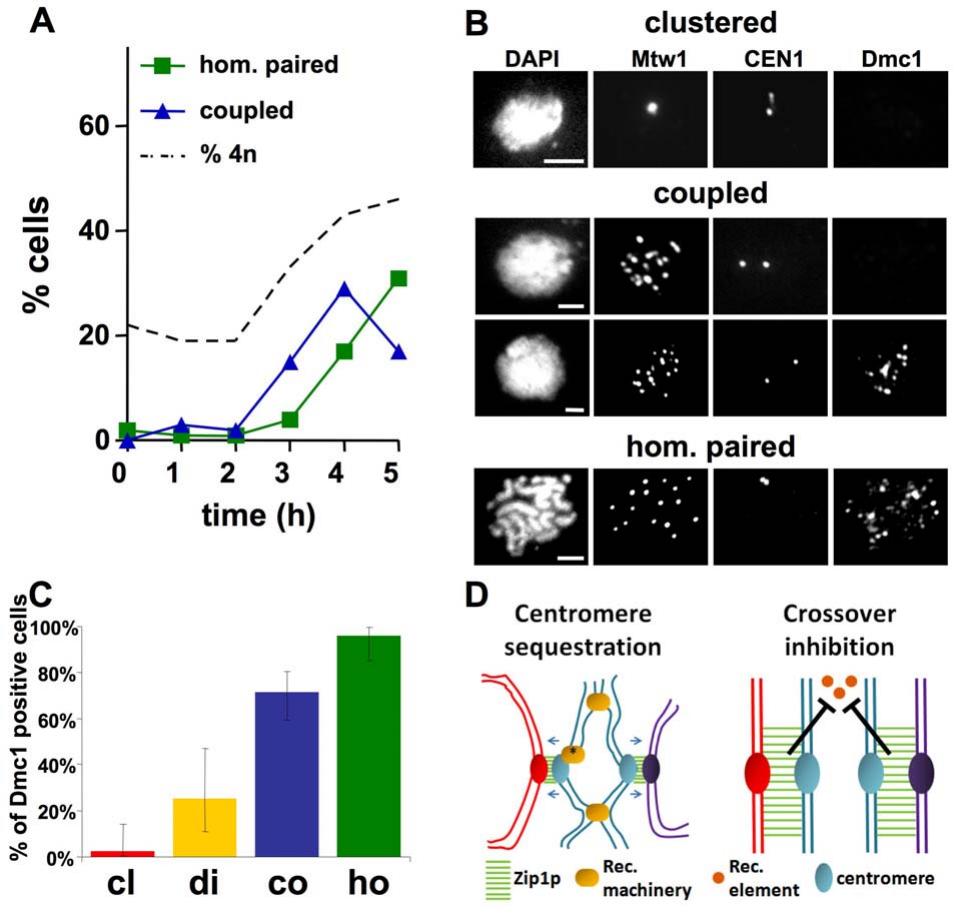
Centromere coupling is co-incident with Dmc1 loading onto the chromosomes. Meiotic cultures were processed to allow evaluation of centromere coupling, and either progression through S phase, or the appearance of Dmc1 on chromosomes. (A) Flow cytometry was used to assay DNA content in wild type cells (DDO45). Parallel samples were evaluated for centromere (Mtw1-13XMYC) organization in chromosome spreads by indirect immunofluorescence. The percentage of 4n cells, and those engaged in centromere coupling or homologous centromere pairing are shown (n>50 at each time point). (B) Dmc1 localization and centromere organization on chromosome spreads prepared from wild type meiotic cells (DDO45) as described in Figure 1. Scale bar: 2µm. (C) Chromosome spreads from wild type cells (DDO45) harvested at three and four hours after induction of meiosis. The graph indicates the percentage of cells in each category that were positive for Dmc1 staining (cl: clustered, n = 41; di: dispersed, n = 20; co: centromeres coupled, n = 69; ho: homologous centromeres paired, n = 48). Error bars: 95% CI. (D) Alternative models of centromere coupling function.

Flow cytometry was used to monitor DNA replication in time course experiments. The emergence of 4n cells coincided with the release of the centromere clustering and the emergence of cells exhibiting centromere coupling (Fig. 4A). The percentage of 4n cells at the final time point roughly equals the number of cells with their centromeres released from the cluster, indicative of entry into the meiotic program (Fig 4A).

Formally, the experiments here do not reveal whether centromeres within the cluster are already organized in couples, though the apparent requirement for Zip1 for coupling and the absence of Zip1 from cells in the clustering stage make this model less appealing. The data do demonstrate that when replication has finished clusters are giving way to dispersed couples. To compare the relative timing of centromere coupling and the early steps of homologous recombination, we asked whether cells involved in coupling have their chromosomes decorated with Dmc1 (Fig. 4B and 4C). Dmc1 loads onto the chromosomes after meiotic DNA double strand breaks (DSBs) are created and prior to chromosomal synapsis, playing a critical role in mediating the formation of early recombination intermediates between homologous chromosomes [22], [23]. Chromosome spreads were first classified into one of the four categories described above (clustered, dispersed, coupled and homologous centromeres paired), and then scored for staining with antibodies against Dmc1.

As expected, cells with clustered centromeres were nearly always negative for Dmc1 staining (Fig. 4B and 4C). Nearly all cells (96%) exhibiting homologous centromere alignment were positive for Dmc1 staining (Fig. 4B and 4C), suggesting that homologous alignment of the *CEN1*'s rarely precedes initiation of homologous recombination. It makes intuitive sense that the alignment of homologous chromosomes would be led by a homology-based recombination process rather than by elements (centromeres) that interact in a homology-independent fashion. When Dmc1 staining was evaluated on cells undergoing coupling, 71% were found to exhibit clear positive staining (Fig. 4B and 4C) suggesting that centromeres are still coupled with non-homologous partners when double strand break repair initiates (Fig. 4D). The fact that 29% of the cells exhibiting centromere coupling were negative for Dmc1 suggests that coupling precedes the loading of Dmc1 onto chromosomes.

## Discussion

Like several other organisms, budding yeast has been shown to exhibit a period in which centromeres become grouped in a homology independent fashion [8]. Zip1 was shown to play a role in this phenomenon [8], [20]. These first studies used a single time point analysis to evaluate centromere-coupling. The analysis revealed key features of the centromere coupling phenomenon in budding yeast, but left unanswered questions related to the timing of the phenomenon, an issue that has been difficult to address in other systems in which homology-independent meiotic centromere interactions have been reported. We have used this approach to directly position centromere coupling in relation to other meiotic events, study its dynamics and better understand the role of Zip1 in this phenomenon.

As demonstrated previously [8], we found that centromere coupling unequivocally takes place before SC formation. The tight centromere clusters that typify cells entering the meiotic program are largely maintained through S-phase and centromeres then transition quickly into non-homologous pairs. It is not clear whether centromeres emerge from the clusters as couples, disperse from the clusters and then become coupled, or both. Individual centromeres clearly do not linger long between clustering and coupling. Cells with these dispersed centromeres were observed, but at very low frequencies. Similarly we do not observe a stage of dispersed centromeres between coupling and homologous pairing. Thus the transition from non-homologous to homologous partners may be brief, or centromere coupling may be dynamic process, as suggested by Tsubouchi and Roeder [8] such that not all the centromeres disengage from their partners simultaneously.

We have studied the dynamics of centromere coupling in *spo11*Δ mutants. We have observed that the process is dramatically affected by the *SPO11* deletion; centromere coupling persists at all times evaluated. Thus, *SPO11* is required, at least indirectly, for the transition from non-homologous coupling to homologous pairing of the centromeres, suggesting a connection between events promoted by Spo11 activity, presumably through the formation of double strand breaks, and a triggered release from coupling after homologous recombination is initiated.

Zip1 is the structural element that holds the lateral elements of the SC together [10]. Zip1 could be imagined to perform a similar role during centromere coupling by bridging the centromeres of non-homologous chromosomes. Our results showing that centromere coupling is abolished at all time points in *zip1* deletion mutants are consistent with this sort of direct structural role for Zip1. However, the formal possibility remains that centromeres still couple in *zip1Δ* cells, but the engagements are short-lived and not detectable with our experimental approach. If Zip1 is the structural element holding centromeres together, Zip1 should localize to the centromeres when they are coupled. We have observed more variable numbers and intensities of punctate Zip1 foci and a lower incidence of Zip1/kinetchore co-localization than previous studies. It seems from these observations and work of others [15], [24] that 1) Zip1 may localize to more than just centromere regions in early meiosis, and 2) that relatively small (undetectable in our assays) amounts of Zip1 may be sufficient to promote coupling.

What is the role of centromere coupling? Centromere coupling has been proposed to promote homologous pairing by holding the centromeres of two chromosomes together while homology at the arms is being assessed [8]. However, *ZIP1* deletion mutants do not have dramatic pairing defects along the chromosome arms [25] and some studies suggest that the bringing together of homologous centromeres is dependent upon interactions between the arms [1], [2]. The results here suggest an alternative possibility; centromere coupling might be preventing the formation of deleterious crossing-over at the centromere. A recent analysis of the global distribution of meiotic crossovers in budding yeast suggested that Zip1 is necessary for the repression of centromeric crossing-over [26]. The authors concluded that Zip1, in some way, directs DSBs repair towards the sister chromatids. We have shown that centromere coupling initiates prior to the association of Dmc1 with the chromosomes and that Dmc1 loading occurs when centromeres are coupled with non-homologous partners. This suggests that Zip1 may be manifesting its role in centromere crossover repression by coupling centromeres with non homologous partners. We propose two different mechanisms by which the coupling process might play this role. First, the sequestration of centromeres with non-homologous partners (Fig. 4D, centromere sequestration) may spatially or topologically prevent interactions between homologous centromere regions, leaving sister chromatids as default partners for repairing DSBs (Fig. 4D, recombination complex marked with an asterisk). Alternatively, (Fig. 4D, crossover inhibition) Zip1 could promote inter-sister over inter-homolog repair of DSBs by blocking the recruitment, or affecting the function, of components of the recombination process that are required for interhomolog events (Fig. 4D, crossover inhibition, orange circles). It has been noted that in early prophase Dmc1 and Zip1 foci are non-overlapping, consistent with the notion that Zip1 deposition may exclude components of the recombination machinery [22]. By this model, centromere coupling might act to increase the polymerization or stability of Zip1 around the centromeres or could be an innocuous by-product of Zip1 assembly properties.

## Materials and Methods

### Yeast strains and culture conditions

All strains were obtained by matings of TSP50 and TSP52, or their isogenic derivatives. We used standard yeast culture methods [27]. To induce meiosis, cells were grown in YP-acetate to 3–4×10^7^ cells per ml, and then shifted to 1% potassium acetate at 10^8^ cells per ml.

### Strain construction

PCR-based methods were used to create complete deletions of ORFs and epitope-tags [28]. Some deletions were created by using PCR to amplify deletion-KANMX insertions from the gene deletion collection (Invitrogen) and these products were then used for transformations. The plasmid pJN2 targeted 256 lacO repeats to *CEN1* (coordinates 153583–154854). Correct integration was confirmed genetically. The P_CYC1_-lacI-GFP cassette was inserted as part of pAFS152, a gift from Aaron Straight. Strain genotypes are reported in Table S1.

### Meiotic chromosome spread preparation

Meiotic nuclear spreads were prepared according to [29] with the following modifications. Cells were spheroplasted using 20 mg per ml zymolyase 100T for approximately 30 minutes. Spheroplasts were briefly suspended in MEM (100mM MES, 10mM EDTA, 500uM MgCl2) containing 1mM PMSF, fixed with 4% paraformaldehyde plus 0.1% Tween20 and spread onto poly-L lysine- coated slides (Fisherbrand Superfrost Plus). Slides were blocked with 4% non-fat dry milk in phosphate buffered saline for at least 30 minutes, and incubated overnight at 40C with primary antibodies. Primary antibodies were mouse anti-Zip1 (a gift from Rebecca Maxfield), rabbit anti-Dmc1p (gift from M. Dresser), rabbit anti-MYC (Bethyl Laboratories A190-105A), mouse anti-MYC, (gift from S. Rankin), chicken anti-GFP (Chemicon AB16901), and rabbit anti-GFP (Invitrogen A11122). Secondary antibodies were Alexa Fluor 488-conjugated goat anti-chicken IgG, Alexa Fluor 546-conjugated goat anti-mouse IgG, and Alexa Fluor 647 conjugated goat anti-rabbit IgG, Alexa Fluor 568-conjugated anti mouse (all from Molecular Probes). Secondary antibody incubations were for two hours at room temperature.

### FACS analysis of DNA replication

FACS analysis and determination of the proportion of 4n cells were performed according to published protocols [30], [31]. Time points during the first five hours of meiosis were analyzed. At the five hour time point, 50% of cells had not entered meiosis (50% of the cells retained clustered centromeres and were negative for Zip1 staining). This strain typically exhibits about 80% asci by 48 hours after the switch to meiosis-inducing medium.

## Supporting Information

Table S1 Yeast strains used in this study.
(0.07 MB DOC)
