# Supplementary material for: Temporal Characterization of Homology-Independent Centromere Coupling in Meiotic Prophase
Source: PLoS One. 2010 Apr 23;5(4):e10336. doi: 10.1371/journal.pone.0010336 (PMC2859069; doi:10.1371/journal.pone.0010336)
Supplement: Table S1 — Yeast strains used in this study. (0.07 MB DOC) [file pone.0010336.s001.doc]

**Table S1: Yeast strains used in this study**

| **Name** | **Genotype** |
| --- | --- |
| TSP50 | *MATα, ura3-13, trp1-63, his3-1, leu2, met13-c, tyr1-2, lys2-2, cyh2-1* |
| TSP52 | *MAT***a***, ura3-13, trp1-63, his3-1, leu2, met13-d, tyr-1, lys2-1, can1* |
| DDO54.3-15b | *MAT***a***, leu2, lys2-2, tyr1-2, met13-c, trp1-Δ63, cyh2-1, his3-Δ1, ura3::[pAFS152:URA3 P_CYC1_-GFP-lacI], CEN1::pJN2[lacO256 LEU2], MTW1-13xMYC-HIS3* |
| DDO54.5-7b | *MAT***a***, leu2, lys2-2, tyr1-2, met13-c, trp1-Δ63, cyh2-1, his3-Δ1, ura3::[pAFS152:URA3 P_CYC1_-GFP-lacI], CEN1::pJN2[lacO256 LEU2], MTW1-13xMYC-HIS3* |
| ABY194-Nd | *MATα, trp1-63, his3-Δ1, leu2, ade1::LYS2, met13-d, lys2-1, ade1::LYS2, ura3::pAFS152[URA3 P_CYC1_-GFP-lacI], CEN1::pJN2[lacO256 LEU2], MTW1-13xMYC-TRP1* |
| DDO50-34c | *MATα, ura3-13, met13-d, trp1-Δ63, leu2, tyr1-1, lys2-1, met13-d, can1-R, ura3::pAFS152[URA3 P_CYC1_-GFP-lacI], CEN1::pJN2[lacO256 LEU2], MTW1-13xMYC-TRP1, zip1::KANMX* |
| DDO51-35b | *MAT***a***, leu2, lys2-2, tyr1-2, met13-c, trp1-Δ63, cyh2-1, his3-Δ1, ura3::[pAFS152:URA3 P_CYC1_-GFP-lacI], CEN1::pJN2[lacO256 LEU2], MTW1-13xMYC-HIS3, zip1::KANMX* |
| DDO50-7d | *MATα, ura3-13, trp1-Δ63, leu2, tyr1-1, lys2-1, met13-d, can1-R, ura3::pAFS152[URA3* *P_CYC1_-GFP-lacI], CEN1::pJN2[lacO256 LEU2], MTW1-13xMYC-TRP1, zip1::KANMX, spo11::KANMX* |
| DDO51-1b | *MAT***a***, leu2, lys2-2, tyr1-2, met13-c, trp1-Δ63, cyh2-1, his3-Δ1, ura3::[pAFS152:URA3 P_CYC1_-GFP-lacI], CEN1::pJN2[lacO256 LEU2], MTW1-13xMYC-HIS3, zip1::KANMX, spo11::URA3* |
| DDO50-21c | *MAT***a***, ura3-13, trp1-Δ63, leu2-?, tyr1-1, lys2-1, met13-d, can1-R, trp1-Δ63, leu2, tyr1-1, lys2-1, met13-d, can1-R, ura3::pAFS152[URA3 P_CYC1_-GFP-lacI], CEN1::pJN2[lacO256 LEU2], MTW1-13xMYC-TRP1, spo11::KANMX* |
| DDO51-22a | *MATα, leu2, lys2-2, tyr1-2, met13-c, trp1-Δ63, cyh2-1, his3-Δ1, ura3::[pAFS152:URA3 P_CYC1_-GFP-lacI], CEN1::pJN2[lacO256 LEU2], MTW1-13xMYC-HIS3, spo11::URA3* |
| DDO45 | (DDO54.5-7b x ABY194-Nd) 2n |
| DDO46 | (DDO54.3- 15b x ABY194-Nd) 2n |
| DDO55 | (DDO50-34c x DDO51-35b) 2n |
| DDO56 | (DDO50-7d x DDO51-1b) 2n |
| DDO60 | (DDO50-21c x DDO51-22a) 2n |
